# Preliminary phytochemical analysis and in vitro antifungal activity of the ethanolic extract of the leaves of *Solanum hispidum* pers. collected in the locality in Obraje - Peru

**DOI:** 10.17843/rpmesp.2022.393.11381

**Published:** 2022-09-30

**Authors:** Jannelle Cyndi Mendoza-León, César Máximo Fuertes Ruitón, Martha Helena Jahuira-Arias

**Affiliations:** 1 Instituto Nacional de Salud - Centro Nacional de Control de Calidad. Lima, Peru. Instituto Nacional de Salud Centro Nacional de Control de Calidad Lima Peru; 2 Universidad Nacional Mayor de San Marcos. Lima, Peru. Universidad Nacional Mayor de San Marcos Universidad Nacional Mayor de San Marcos Lima Peru

**Keywords:** Antifungical, *in vitro*, phytochemical, plant extracts

## Abstract

**Objective.:**

To analyze and determine the in vitro antifungical activity of the ethanolic extract of the leaves of Solanum hispidum Pers.

**Materials and methods.:**

We carried out a preliminary qualitative phytochemical analysis by color and precipitation reactions. We evaluated the in vitro antifungical activity against Candida albicans, Aspergillus brasilensis and Trichophyton mentagrophytes by using the agar well diffusion method and the minimum inhibitory concentration (MIC) assay.

**Results.:**

Preliminary qualitative phytochemical analysis showed the presence of phenolic compounds, tannins, flavonoids, steroids, alkaloids and saponins. In vitro antifungal activity was demonstrated for all fungal cultures with inhibition halos between 23 to 26 mm. The MIC values were 125, 250, and 125 μg/mL for C. albicans, A. brasilensis, and T. mentagrophytes, respectively.

**Conclusions.:**

The ethanolic extract of the leaves of Solanum hispidum Pers. contains important secondary metabolites and has moderate antifungical activity.

## INTRODUCTION

Plant extracts are widely used in the treatment of diseases, particularly as antifungals. Currently, research focused on their active biological components is promising, and the World Health Organization (WHO) has proposed that traditional medicine be considered for inclusion in the health care system ^1^.

Dermatomycosis is one of the most frequent skin diseases, and there are numerous epidemiological studies in our country on its incidence in the population ^2^. Drug resistance, therapeutic failures, adverse effects, and toxicity regarding the use of conventional antifungal drugs represent a problem, so it is necessary to seek new alternatives for treatment ^3^. Traditional medicine is an important option; however, it needs to be scientifically validated towards conventional medicine.

The *Solanaceae* family is one of the most diverse and the genus *Solanum* is widely distributed in Peru; *Solanum hispidum* Pers is found between 2500 to 3500 m altitude ^4^. This plant grows abundantly in Carhuaz, where it is known as *ñahui pashta* and is traditionally used by the local population to treat foot mycosis by topical application of the fruit contents ^5^.

Previous studies have demonstrated the *in vitro* antifungal activity of several species of the genus *Solanum*, such as *Solanum crysotrichum* against pathogens such as *Trichophyton mentagrophytes*, *Trichophyton rubrum* and *Trichophyton gypseum* ^6^. Subsequently, clinical studies were conducted on a topical solution derived from the methanolic extract of its leaves, which showed effectiveness against *Tinea pedis* ^7^. *Solanum melongena* showed antifungal activity against *Trichophyton mentagrophytes*, *Trichophyton rubrum*, *Trichopyton tonsurans*, *Candida albicans* and *Trichosporon beigeii* ^8^. In addition, it has been reported that *Solanum xanthocarpum* inhibits the growth of *Aspergillus fumigatus*, *Aspergillus flavus* and *Aspergillus niger* ^9^; other recent studies also demonstrated antifungal activity against *Candida albicans* ^10^. The species *Solanum nigrum* L has antifungal activity against *Trichophyton rubrum*, *Trichophyton tonsurans*, *Trichophyton mentagrophytes*, *Microsporum gypseum* and *Candida albicans* ^11^; alkaloids, flavonoids, coumarins, tannins and saponins were found among its phytochemical compounds ^12^.

The leaves of *Solanum hispidum* Pers are used as an antifungal in Mexican folk medicine, and its antifungal activity against *Trichophyton mentagrophytes*, *Trichophyton rubrum*, *Aspergillus niger* and *Candida albicans* has been demonstrated; the strains that showed greater sensitivity were *Trichophyton mentagrophytes* and *Trichophyton rubrum*; in addition, steroidal saponins were identified and isolated ^13^. On the other hand, the recent study by Retamozo ^14^ reported the abundance of steroidal glycoalkaloids as main secondary metabolites in leaves and fruits of this species. However, there is no study in Peru on the evaluation of their properties against fungal agents. Therefore, this study aims at the preliminary phytochemical analysis and antifungal activity of the ethanolic extract of leaves of *Solanum hispidum* Pers.

**KEY MESSAGES**

Motivation for the study: this study seeks to validate the ethnobotanical use of *Solanum hispidum* Pers as an antifungal, as well as to evaluate its phytochemistry in order to determine the main metabolites and demonstrate its *in vitro* activity against different fungal agents.

Main findings: we mainly found steroids and alkaloids in the extract, as well as moderate antifungal activity against *C. albicans* ATCC 10231, *A. brasilensis* ATCC 16404 and *T. mentagrophytes* ATCC 9533.

Implications: it is necessary to continue researching this topic, with the purpose of obtaining topical and affordable phytopharmaceuticals with antifungal activity.

## MATERIALS AND METHODS

### Collection of plant material

**Figure 1:**
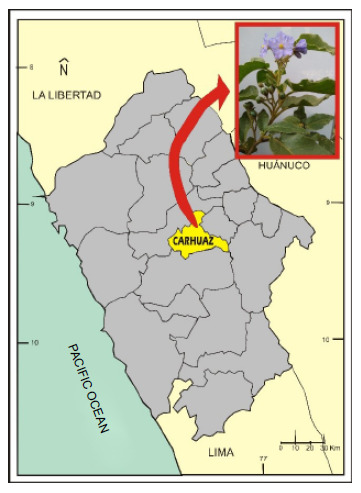
Location where the leaves of *Solanum hispidum* Pers were collected.

Fresh leaves of *Solanum hispidum* Pers were randomly collected from different specimens distributed in the department of Ancash, province of Carhuaz, district of Acopampa, locality of Obraje, an altitude of 2750 m (Figure 1).

The species was taxonomically identified and certified by the San Marcos herbarium of the Natural History Museum of the Universidad Nacional Mayor de San Marcos (code: 053-USM-2017).

### Obtaining the ethanolic extract of the plant

The leaves of *Solanum hispidum* Pers were washed with distilled water and initially dried at room temperature for seven days, the drying process was then completed at 40 °C in an oven with circulating air for five days; subsequently, the leaves were crushed and ground until a uniform fine powder was obtained ^15^.

The powder was mixed with 90% ethanol in a 1:10 ratio in an amber glass bottle which was kept at room temperature for seven days with frequent manual shaking. Then, the extract was filtered using gauze and 20 µm cellulose filter paper; subsequently, the solvent was evaporated under reduced pressure in a rotary evaporator® (Buchi-R-100) at 40 °C and 60 rpm ^15^ ^,^ ^16^. The dried extract was stored refrigerated at 2 to 8 °C until use.

### Solubility test

To 20 mg of the stabilized ethanolic extract of *Solanum hispidum* Pers we added 1 mL of each of the following solvents: distilled water, ethanol, methanol, ethyl acetate, chloroform, diethyl ether and n-hexane. Then each tube was shaken and the results were observed for a maximum of 10 min ^17^.

### Phytochemical analysis

To determine the presence or absence of the main secondary metabolites, we carried out qualitative staining and precipitation tests using the standard chemical methods described by Lock ^18^.

### Microorganisms

We used strains of *Candida albicans* ATCC 10231, *Trichophyton mentagrophytes* ATCC 9533 and *Aspergillus brasilensis* ATCC 16404 provided by the Cepario of the Microbiology and Biological Laboratory of the Instituto Nacional de Salud. 

*T. mentagrophytes* and *A. brasilensis* strains were grown on Sabouraud dextrose agar (ADS) for 7 and 10 days, respectively. *C. albicans* was incubated in Sabouraud dextrose broth for 48 h, the incubation temperature was 20 to 25 °C. The strains were suspended and adjusted with a spectrophotometer to a concentration of 1 x 10^6^ CFU/mL for *C. albicans* and 1 x 10^5^ CFU/mL for *T. mentagrophytes* and *A. brasiliensis*, respectively ^19^.

### *In vitro* antifungal activity

The antifungal activity of the ethanolic extract of *Solanum hispidum* Pers leaves was demonstrated using the agar well diffusion method ^19^.

We inoculated 1 mL of the fungal suspension (0.5 x 10^5^ CFU/mL for *C. albicans* and 0.5 x 104 CFU/mL for *T. mentagrophytes* and *A. brasiliensis*) into 20 mL of Sabouraud dextrose agar (ADS). It was mixed uniformly and poured homogeneously into Petri dishes, then when the surface solidified, 11 mm diameter wells were punched with a sterile stainless-steel punch; 100 µL of the ethanolic extract (25 mg/mL) was added to each well ^19^. Subsequently, the plates were incubated at 37 °C for 24 h for *C. albicans*, 72 h for *A. brasiliensis*, and seven days for *T. mentagrophytes*; dimethyl sulfoxide (DMSO) and distilled water were used as negative controls ^20^ ^,^ ^21^.

We evaluated the antifungal activity after the incubation time was finished by measuring the diameter of the inhibition zone in mm. The antifungal activity of the extract was evaluated by comparing the inhibition zones with standard antifungals for each microorganism (nystatin at 0.2 mg/mL ketoconazole at 0.2 mg/mL and fluconazole at 0.2 mg/mL) ^19^. Eight replicates were carried out for each strain.

### Determination of the minimum inhibitory concentration (MIC)

For the determination of the minimum inhibitory concentration (MIC), we used the colorimetric microdilution method in microplate following the Clinical and Laboratory Standards Institute (CLSI) protocols ^22^ ^,^ ^23^ modified by Liu ^24^ and Fernandez ^20^.

We obtained suspensions in RPMI 1640 (Sigma-Aldrich) with resazurin for each strain: ranges of 0.5 - 2.5 x 10^3^ CFU/mL for *C. albicans* and ranges of 0.6 to 3 x 10^4^ CFU/mL for *A. brasiliensis* and T. mentagrophytes, respectively. In addition, serial dilutions of ethanolic extract of *Solanum hispidum* Pers leaves were prepared in RPMI 1640 medium (sigma-Aldrich) with resazurin, the evaluated concentrations ranged from 3.91 to 2000 µg/mL. Each assay was carried out in triplicate for each strain. Plates were incubated aerobically at 37 °C for 24 h for *C. albicans*, five days for *A. brasiliensis* and seven days for *T. mentagrophytes*. We visually evaluated the results after the incubation period was over; when the biological activity was inhibited the original color decreased noticeably ^24^.

In all assays, the antifungals ketoconazole and fluconazole in RPMI medium ^22^ ^,^ ^23^ with resazurin 0.05 mg/mL and sterility controls containing the culture medium with resazurin 0.05 mg/mL without the microorganism were used as positive controls ^21^.

For the interpretation of antifungal activity, we used the qualitative criteria described by Holets *et al*. (2002) ^25^, i.e., MIC < 100 µg/ML (good), 100 to < 500 µg/mL (moderate), 500 to 1000 µg/mL (weak).

### Statistical analysis

The data obtained were analyzed using MINITAB 19 software. We carried out the descriptive and statistical analysis of the variables. 

### Ethical aspects

This project was approved by the Institutional Research Ethics Committee of the Instituto Nacional de Salud (CIEI -INS), RD No. 533-2019 OGITT/INS. We used strains from the (ATCC) maintained at the Microbiology and Biological Laboratory of the Instituto Nacional de Salud at -70 ºC. No patients were involved in this study.

## RESULTS

### Solubility test

The solubility tests results are described in Table 1, which shows that the stabilized ethanolic extract of *Solanum hispidum* Pers leaves was poorly soluble (+) in the solvent n-hexane; soluble (++) in distilled water, ethyl acetate, chloroform, diethyl ether; and very soluble (+++) in alcoholic solvents such as ethanol and methanol. The decrease in solubility was directly proportional to the polarity index of the test solvent.

**Table 1:** Solubility of the stabilized ethanolic extract of *Solanum hispidum* Pers.

Solvent	Polarity index	Result
Distilled water	9.0	++
Ethanol	5.2	+++
Methanol	5.1	+++
Ethyl acetate	4.4	++
Chloroform	4.1	++
Diethyl ether	2.8	++
n-hexane	0.0	+

-: insoluble; +: slightly soluble; ++: soluble; +++: very soluble

### Preliminary phytochemical analysis

The ethanolic extract obtained from leaves of *Solanum hispidum* Pers showed a variety of secondary metabolites; we identified phenolic compounds, tannins, flavonoids, steroids, alkaloids and saponins (Table 2).

**Table 2:** Secondary metabolites identified in the ethanolic extract of *Solanum hispidum* Pers.

Metabolite	Test	Result
Phenolic compounds	Ferric trichloride	+
Tannins	Gelatin - NaCl	+
Flavonoids	Shinoda	+
	Pews	+
	Sodium hydroxide	+
Steroids	Trichloroacetic Acid	+
	Liebermann-Burchard	+
	Rosenthaler	+
	Salkowski	+
Alkaloids	Draggendorf	+
	Mayer	+
	Bouchardat	+
	Sonneschein	+
Saponins	Foam	+
Coumarins	Hidroxilamina	-
Naphthoquinones	Bornträger	-
Anthraquinones	Bornträger	-
Cardiotonic glycosides	Kedde	-
Sesquiterpene lactones	Bajlet	-
Leucoanthocyanins	Rosenheim	-

- : absence of the metabolite, +: presence of the metabolite

Coumarins, naphthoquinones, anthraquinones, cardiotonic glycosides, sesquiterpene lactones and leucoanthocyanins were not found in the ethanolic extract.

### *In vitro* antifungal activity

**Figure 2:**
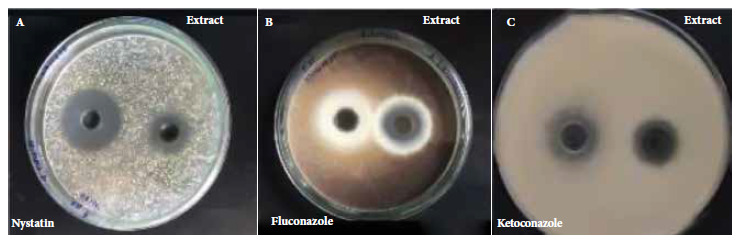
Representative experiment showing the comparison of the inhibition halos of each microorganism: A) *Candida albicans* ATCC 10231, B) *Aspergillus brasiliensis* ATCC 16404 and C) *Trichophyton mentagrophytes* ATCC 9533 against ethanolic extract of *Solanum hispidum* Pers leaves and standard antifungals (positive controls).

*In vitro* antifungal activity was evaluated by the agar culture diffusion method against *Candida albicans* ATCC 10231, *Aspergillus brasiliensis* ATCC 16404 and *Trichophyton mentagrophytes* ATCC 9533; the activity was demonstrated by inhibition halos (Figure 2).

Table 3 presents the results of the antifungal activity of the leaves of *Solanum hispidum* Pers. The assay showed that the inhibition halo was 26 mm (± 0.38) for *C. albicans*, 23 mm (± 0.53) for *A. brasiliensis* and 25 mm (± 1.31) for T*. mentagrophytes*. The ethanolic extract of *Solanum hispidum* Pers leaves showed greater activity against *C. albicans*, however, its positive control (nystatin) presented a larger halo (30 mm) compared to the ethanolic extract.

**Table 3:** Antifungal activity of the ethanolic extract of *Solanum hispidum* Pers leaves against microorganisms.

Microorganism	Ethanolic extract (mg/mL)	Inhibition halo diameter mm ±SD
*Candida albicans* ATCC 10231	25 mg/mL Nystatin (2mg/mL)	26 ± 0.38 30 ± 0.38
*Aspergillus brasiliensis* ATCC 16404	25 mg/mL Fluconazole (2mg/mL)	23 ± 0.53 15 ± 0.53
*Trichophyton mentagrophytes* ATCC 9533	25 mg/mL Ketonazole (2mg/mL)	25 ± 1.31 24 ± 0.76

SD: standard deviation

### Determination of the minimum inhibitory concentration (MIC)

Following the demonstration of antifungal activity, we evaluated the MIC. The results showed that the lowest concentration of the ethanolic extract of *Solanum hispidum* Pers leaves that completely inhibits growth of *C. albicans* was 125 µg/mL; for *A. brasiliensis* it was 250 µg/mL and for *T. mentagrophytes* it was 125 µg/mL; based on the criteria of antifungal activity, all of them showed moderate activity (Table 4).

**Table 4:** Minimum inhibitory concentration (MIC) values (µg/mL) of the ethanolic extract of *Solanum hispidum* Pers leaves against microorganisms.

Microorganism	Minimum inhibitory concentration (µg/mL)	Antifungal activity criteria
*Candida albicans* ATCC 10231	125	Moderate
*Aspergillus brasiliensis* ATCC 16404	250	Moderate
*Trichophyton mentagrophytes* ATCC 9533	125	Moderate

## DISCUSSION

This study determined that the stabilized ethanolic extract of *Solanum hispidum* Pers leaves presents a higher solubility against ethanol and methanol, that is, with tendency to polar solvents, both of which are widely used; however, in this study we continued with ethanol due to its availability, considering, in addition, that most metabolites with antifungal activity have intermediate polarity and can be easily concentrated in this type of solvents ^26^. Our results agree with other studies carried out on crude extracts ^24^.

Qualitative analyses were carried out in order to detect the metabolites present in the ethanolic extract of *Solanum hispidum* Pers leaves. Our results showed the presence of multiple metabolites such as phenolic compounds, tannins, flavonoids, steroids, alkaloids and saponins; in addition, to verify their presence, we used four different tests for alkaloids and four differential tests for steroids with respect to triterpenoids ^18^, thus demonstrating the reliability of the results.

In this sense, the genus Solanum has been reported to have an abundance of alkaloids and steroids, such is the case of *Solanum chrysotrichum* ^6^ ^,^ ^7^, *Solanum xanthocarpum* ^27^, *Solanum nigrum* ^11^, *Solanum surattense* ^28^ and *Solanum quitoense* ^29^. Retamozo ^14^ was able to identify steroidal glycoalkaloids through qualitative quantitative tests in the same species, *Solanum hispidum* Pers; in addition, he analyzed the content of leaves and fruits, demonstrating a higher content in the fruit compared to the leaves; this also explained that the variability of the content is influenced by different factors such as vegetative state, time of collection, origin, etc. This confirms the presence of alkaloids and steroids as components of *Solanum hispidum* Pers. leaves extract.

We used a concentration of 25 mg/mL of the extract in DMSO, based on previous screening studies of ethanolic extracts of Peruvian plants with antifungal activity; Rojas *et al*. ^19^ and Quiroz ^21^ obtained favorable results using this concentration in 24 and 8 medicinal plants, respectively.

Regarding antifungal activity, the ethanol extract of *Solanum* hispidum Pers leaves showed an inhibitory effect on the growth of *C. albicans*, *A. brasiliensis* and T. mentagrophytes with inhibition zones between 23 and 26 mm. In this sense, Rojas *et al*. ^19^ mentioned that antifungal activity with inhibition halos greater than 18 mm, using the agar well diffusion method, is an indicator of good performance as a potential therapeutic agent.

The research carried out by Das *et al*. ^8^ demonstrated halos of 18 mm against the pathogen *C. albicans* using *Solanum melongena*; on the other hand, Shubha *et al*. ^27^ reported halos of 12 mm using *Solanum xanthocarpum* extract; both species belong to the *Solanaceae* family. In previous studies, *Solanum nigrum* and *Solanum xanthocarpum* species also showed activity against *C. albicans* by the disk diffusion method ^26^. In addition, a screening study of Peruvian plants revealed that a species of the *Solanaceae* family showed higher activity, evidencing inhibition zones of 19 mm with the agar well diffusion method ^19^. The results of this research show higher inhibition halos (≥ 23 mm) compared to studies against other species of the genus *Solanum* ^8^ ^,^ ^19^ ^,^ ^26^ ^,^ ^27^.

Also, our study showed moderate antifungal activity for *C. albicans* with values of 125 µg/mL, which are lower than the 256 µg/mL reported for *Solanum mammosum* species ^30^. Antifungal activity has also been reported against other fungal species such as *Aspergillus* sp or *Solanum xanthocarpum* against *A. niger* ^10^, showing antifungal activity with MIC of 250 µg/mL, our study also found this same value (250 µg/mL) against *A. brasiliensis*. For *Trichophyton mentagrophytes* we found a MIC of 125 µg/mL; similar results have been reported with *Solanum mammosum* with values of 256 µg/mL ^30^ for *T. mentagrofphytes*.

Many species of the *Solanaceae* family such as *Solanum chrysotrichum* ^6^ ^,^ ^7^, *Solanum melogena* ^8^, *Solanum nigrum* ^11^, *Solanum xanthocarpum* ^23^ and *Solanum mammosum* ^30^ also showed antifungal activity, which may be due to the presence of saponins, alkaloids, steroids and/or flavonoids, which may act individually or synergistically by a mechanism of action that remains unknown.

Both steroids and alkaloids have high biological activity and are a group of cyclic compounds that have been studied due to their antimicrobial effects, which has been confirmed through the isolation of bioactive compounds with potent *in vitro* antifungal activity ^30^.

One of the limitations of our study is that we used only the leaves and not the fruits, which are also used in the surrounding areas where the specimens were collected. This is due to the fact that they are not renewable and it was preferred not to affect their natural and habitual growth; nevertheless, we propose that the next stage, in order to complement this article, is to study the fractionation and characterization of the bioactive compounds of these fruits.

This *in vitro* study presents a preliminary phytochemical analysis of *Solanum hispidum* Pers. extract, in which it was possible to identify the main secondary metabolites, information not previously known, and to demonstrate the moderate *in vitro* antifungal activity of the ethanolic extract of *Solanum hispidum* Pers. leaves.
