# Supplementary material for: Preliminary phytochemical analysis and in vitro antifungal activity of the ethanolic extract of the leaves of Solanum hispidum pers. collected in the locality in Obraje - Peru
Source: Rev Peru Med Exp Salud Publica. 2022 Sep 30;39(3):321–7. doi: 10.17843/rpmesp.2022.393.11381 (PMC11397664; doi:10.17843/rpmesp.2022.393.11381)
Supplement: Translation

# Translation: Análisis fitoquímico preliminar y actividad antifúngica In vitro del extracto etanólico de las hojas de *Solanum hispidum *pers. Colectadas en la localidad Obraje - Perú

**Authors:** Jannelle Cyndi Mendoza-León, César Máximo Fuertes Ruitón, Martha Helena Jahuira-Arias

**Affiliations:** 1 Instituto Nacional de Salud - Centro Nacional de Control de Calidad, Lima, Perú.; 2 Universidad Nacional Mayor de San Marcos, Lima, Perú.

**Keywords:** Antifúngicos, extractos vegetales, fitoquímicos, *in vitro*

## Abstract

**Objetivo.:**

Analizar y determinar la actividad antifúngica *in vitro* del extracto etanólico de las hojas de *Solanum hispidum* Pers.

**Materiales y métodos.:**

Se realizó el análisis fitoquímico preliminar cualitativo mediante reacciones de color y precipitación. Se investigó la actividad antifúngica *in vitro* frente a *Candida albicans*, *Aspergillus brasilensis* y *Trichophyton mentagrophytes* usando el método de difusión en pozo de agar y el ensayo de la concentración mínima inhibitoria (CMI).

**Resultados.:**

El análisis fitoquímico preliminar cualitativo mostró la presencia de compuestos fenólicos, taninos, flavonoides, esteroides, alcaloides y saponinas. La actividad antifúngica *in vitro* fue demostrada para todos cultivos fúngicos con halos de inhibición entre 23 a 26 mm. Los valores de la CMI fueron de 125, 250 y 125 µg/mL para *C. albicans*, *A. brasilensis* y *T. mentagrophytes,* respectivamente.

**Conclusiones.:**

El extracto etanólico de las hojas de *Solanum hispidum* Pers contiene importantes metabolitos secundarios y tiene moderada actividad antifúngica.

## INTRODUCCIÓN

Los extractos vegetales son ampliamente usados en el tratamiento de enfermedades, particularmente como antifúngicos. Actualmente, las investigaciones enfocadas en sus componentes biológicos activos son prometedoras; asimismo, la Organización Mundial de la Salud (OMS) ha propuesto que se considere la inclusión de la medicina tradicional en el sistema de atención de la salud ^1^.

Las dermatomicosis constituyen una de las enfermedades cutáneas más frecuentes, los estudios epidemiológicos en nuestro país sobre la incidencia en la población son numerosos ^2^. La resistencia a los fármacos, los fracasos terapéuticos, los efectos adversos y la toxicidad que existe con el uso de antimicóticos convencionales representan un problema, por lo que es necesario buscar nuevas alternativas para el tratamiento ^3^. La medicina tradicional representa una opción importante, sin embargo, necesita ser validada científicamente hacia la medicina convencional.

La familia *Solanaceae* es una de las más diversas y el género *Solanum* está ampliamente distribuido en el Perú; *Solanum hispidum* Pers se encuentra entre 2500 a 3500 m de altitud ^4^. Esta planta crece abundantemente en Carhuaz, donde se le conoce como *ñahui pashta* y es tradicionalmente usada por la población local para el tratamiento de micosis de los pies mediante aplicación tópica del contenido de los frutos^ (^ ^5^.

Estudios previos han demostrado la actividad antifúngica *in vitro* de varias especies del género *Solanum*, como *Solanum crysotrichum* para patógenos como *Trichophyton mentagrophytes*, *Trichophyton rubrum* y *Trichophyton gypseum* ^6^. Posteriormente, se realizaron estudios clínicos en una crema derivada del extracto metanólico de sus hojas, la cual mostró efectividad en *Tinea pedís* ^(^ ^7^. *Solanum melongena* demostró actividad antifúngica contra *Trichophyton mentagrophytes, Trichophyton rubrum, Trichopyton tonsurans, Candida albicans *y *Trichosporon beigeii* ^8^. Además, se evidenció que *Solanum xanthocarpum* inhibe el crecimiento de *Aspergillus fumigatus*, A*spergillus flavus* y *Aspergillus niger* ^9^, otros estudios recientes demostraron también la actividad antifúngica frente a *Candida albicans* ^10^. La especie *Solanum nigrum* L posee actividad antifúngica contra *Trichophyton rubrum*, *Trichophyton tonsurans*, *Trichophyton mentagrophytes*, *Microsporum gypseum* y *Candida albicans* ^11^; y entre sus compuestos fitoquímicos se encontraron alcaloides, flavonoides, cumarinas, taninos y saponinas ^12^. 

Las hojas de *Solanum hispidum* Pers son usadas como antimicótico en la medicina folclórica mexicana, y se demostró su actividad antifúngica contra *Trichophyton mentagrophytes, Trichophyton rubrum*, *Aspergillus niger* y *Candida albicans*; las cepas que mostraron mayor sensibilidad fueron *Trichophyton mentagrophytes* y *Trichophyton rubrum*; además, se determinaron y aislaron saponinas esteroidales ^13^. Por otra parte, el estudio reciente de Retamozo ^14^ reportó la abundancia de glicoalcaloides esteroidales como principales metabolitos secundarios en hojas y frutos de esta especie. Sin embargo, no existe ningún estudio en Perú sobre la evaluación de sus propiedades frente a agentes fúngicos. Por lo tanto, el presente estudio tiene como objetivo el análisis fitoquímico preliminar y actividad antifúngica del extracto etanólico de las hojas de *Solanum hispidum* Pers.

**MENSAJE CLAVE**

Motivación para realizar el estudio: este estudio busca validar el uso etnobotánico de *Solanum hispidum* Pers como antifúngico, así como contribuir a evaluar su fitoquímica para poder determinar los principales metabolitos y demostrar la actividad in vitro frente a diferentes agentes fúngicos.

Principales hallazgos: se evidenció la presencia principal de esteroides y alcaloides en el extracto, así como la actividad antifúngica moderada frente a *C. albicans *ATCC 10231, *A. brasilensis *ATCC 16404 y *T. mentagrophytes *ATCC 9533.

Implicancias: es necesario que se continúe investigando este tema, con el propósito de obtener la formulación de fitofármacos de aplicación tópica con actividad antifúngica, y que, además, sean asequibles.

## MATERIALES Y MÉTODOS

### Colección del material vegetal

Se colectaron al azar hojas frescas de *Solanum hispidum* Pers de diferentes ejemplares distribuidos en el departamento de Ancash, provincia de Carhuaz, distrito de Acopampa, localidad de Obraje. Ubicada a una altitud de 2750 m de altitud (Figura 1).

**Figura 1:** Ubicación del lugar de colecta de hojas de *Solanum hispidum *Pers.

La especie fue identificada taxonómicamente y certificada por el herbario San Marcos del Museo de Historia Natural de la Universidad Nacional Mayor de San Marcos (código: 053-USM-2017).

### Obtención del extracto etanólico de la planta

Las hojas de *Solanum hispidum* Pers fueron lavadas con agua destilada e inicialmente secadas a temperatura ambiente durante siete días, luego se completó el secado a 40 °C en estufa con aire circulante por cinco días; posteriormente, las hojas se trituraron y molieron hasta obtener un polvo fino uniforme ^15^.

El polvo fue mezclado con etanol al 90% en una proporción 1:10 en un frasco de vidrio ámbar el cual se mantuvo a temperatura ambiente durante siete días con agitación manual frecuente. Luego, el extracto fue filtrado empleando una gasa y papel filtro de celulosa 20 µm; posteriormente, el solvente se evaporó bajo presión reducida en un rotaevaporador® (Buchi- R-100) a 40 °C y 60 rpm ^15^ ^,^ ^16^. El extracto seco se almacenó en refrigeración entre 2 a 8 ºC hasta su uso.

### Prueba de solubilidad

Se colocó 20 mg del extracto etanólico estabilizado de *Solanum hispidum* Pers y se agregó 1 mL de cada uno de los siguientes solventes: agua destilada, etanol, metanol, acetato de etilo, cloroformo, éter dietílico y n-hexano. Luego se agitó cada tubo y se procedió a observar los resultados por un máximo de 10 min ^17^.

### Análisis fitoquímico

Para determinar la presencia o ausencia de los principales metabolitos secundarios se realizaron las pruebas cualitativas de coloración y precipitación mediante los métodos químicos estándar descritos por Lock ^18^.

### Microorganismos

Se emplearon cepas de *Candida albicans* ATCC 10231, *Trichophyton mentagrophytes* ATCC 9533 y *Aspergillus brasilensis* ATCC 16404 provistos por el Cepario del Laboratorio de Microbiología y Biológicos del Instituto Nacional de Salud.

Las cepas de *T. mentagrophytes* y *A. brasilensis* fueron cultivados en agar dextrosa Sabouraud (ADS) durante 7 y 10 días, respectivamente.* C. albicans *se incubó en caldo dextrosa Sabouraud por 48 h, la temperatura de incubación fue 20 a 25 ºC. Las cepas se suspendieron y ajustaron con un espectofotómetro hasta lograr una concentración de 1 x 10^6^ UFC/mL para *C. albicans *y 1 x 10^5^ UFC/mL para* T. mentagrophytes* y *A. brasiliensis,* respectivamente ^19^.

### Actividad antifúngica* in vitro*

La actividad antifúngica del extracto etanólico de las hojas de *Solanum hispidum* Pers se realizó utilizando el método de difusión en pozos de agar ^19^.

Se inoculó en 20 mL de agar dextrosa Sabouraud (ADS) 1 mL de la suspensión fúngica (0,5 x 10^5^ UFC/mL para *C. albicans* y 0,5 x 10^4^ UFC/mL para *T. mentagrophytes* y *A. brasiliensis*). Se mezcló uniformemente y se vertió homogéneamente en placas Petri, luego cuando la superficie se solidificó se perforó pozos de 11 mm de diámetro con un perforador de acero inoxidable estéril; se agregó 100 µL del extracto etanólico (25 mg/mL) a cada un pozo ^19^. Posteriormente, las placas se incubaron a 37 °C por 24 h para *C. albicans*, 72 h para *A. brasiliensis* y siete días para *T. mentagrophytes*; como control negativo se empleó dimetilsulfóxido (DMSO) y agua destilada ^20^ ^,^ ^21^.

Al finalizar el tiempo de incubación, se evaluó la actividad antifúngica midiendo el diámetro de la zona de inhibición en mm. La actividad antifúngica del extracto fue evaluada comparando las zonas de inhibición con antifúngicos estándar para cada microorganismo (nistatina a 0,2 mg/mL ketoconazol a 0,2 mg/mL y fluconazol a 0,2 mg/mL) ^19^. Se realizaron ocho réplicas por cada cepa.

### Determinación de la concentración mínima inhibitoria (CMI)

Para la determinación de la concentración mínima inhibitoria (CMI) se utilizó el método de microdilución colorimétrico en microplaca siguiendo los protocolos del Instituto de Estándares Clínicos y de Laboratorio (CLSI) ^22^ ^,^ ^23^ modificado por Liu ^24^ y Fernández ^20^.

Se obtuvieron suspensiones en RPMI 1640 (Sigma-Aldrich) con resazurina para cada cepa: rangos de 0,5 - 2,5 x 10^3^ UFC/mL para *C. albicans *y rangos de 0,6 a 3 x 10^4^ UFC/mL para *A. brasiliensis* y *T. mentagrophytes*. Además, se prepararon diluciones seriadas del extracto etanólico de las hojas de *Solanum hispidum* Pers en medio RPMI 1640 (sigma-Aldrich) con resazurina, las concentraciones evaluadas estuvieron en rangos de 3,91 a 2000 µg/mL. Cada ensayo fue realizado por triplicado para cada cepa. Las placas se incubaron aeróbicamente a 37 ºC por 24 h para *C. albicans*, cinco días para A*. brasiliensis* y siete días para *T. mentagrophytes*. Culminado el tiempo de incubación se realizó la evaluación visual, cuando existe inhibición de la actividad biológica se reduce marcadamente el color original ^24^.

En todos los ensayos se emplearon como controles positivos los antifúngicos ketoconazol y fluconazol en medio RPMI ^22^ ^,^ ^23^ con resazurina 0,05 mg/mL y controles de esterilidad que contenían el medio de cultivo con resazurina 0,05 mg/mL sin el microorganismo ^21^.

Para la interpretación de la actividad antifúngica se empleó los criterios cualitativos descritos por Holets *et al.* (2002) ^25^, es decir: CMI < 100 µg/Ml (buena), 100 a < 500 µg/mL (moderada), 500 a 1000 µg/mL (débil).

### Análisis estadístico

Los datos obtenidos fueron analizados a través del *software* de MINITAB 19. Se realizó análisis estadístico descriptivo de las variables. 

### Aspectos éticos

Este proyecto fue aprobado por el Comité institucional de ética en investigación del Instituto Nacional de Salud (CIEI -INS), RD N.º 533-2019 OGITT/INS. Se utilizó cepas de la (ATCC) mantenidas en el Laboratorio de Microbiología y Biológicos del Instituto Nacional de Salud a -70 ºC. En este estudio no se involucraron pacientes.

## RESULTADOS

### Prueba de solubilidad

Las pruebas de solubilidad describen los resultados en la Tabla 1, en la cual se evidencia que el extracto etanólico estabilizado de las hojas de *Solanum hispidum* Pers resultó poco soluble (+) en el solvente n-hexano; soluble (++) en agua destilada, acetato de etilo, cloroformo, éter dietílico; muy solube (+++) en solventes alcohólicos como el etanol y metanol. La disminución de la solubilidad fue directamente proporcional al índice de polaridad del solvente de prueba.

**Tabla 1:** Solubilidad del extracto etanólico estabilizado de *Solanum hispidum *Pers.

Solvente	Índice de polaridad	Resultado
Agua destilada	9,0	++
Etanol	5,2	+++
Metanol	5,1	+++
Acetato de etilo	4,4	++
Cloroformo	4,1	++
Éter dietílico	2,8	++
n-hexano	0,0	+

-: Insoluble; +: poco soluble; ++: soluble; +++: muy soluble

### Análisis fitoquímico preliminar

El extracto etanólico obtenido a partir de hojas de *Solanum hispidum* Pers presentó una variedad de metabolitos secundarios; se logró identificar compuestos fenólicos, taninos, flavonoides, esteroides, alcaloides y saponinas (Tabla 2).

**Tabla 2:** Metabolitos secundarios identificados en el extracto etanólico de *Solanum hispidum *Pers.

Metabolito	Test	Resultado
Compuestos fenólicos	Tricloruro férrico	+
Taninos	Gelatina - NaCl	+
Flavonoides	Shinoda	+
	Pews	+
	Hidróxido de sodio	+
Esteroides	Ácido Tricloroacético	+
	Liebermann-Burchard	+
	Rosenthaler	+
	Salkowski	+
Alcaloides	Draggendorf	+
	Mayer	+
	Bouchardat	+
	Sonneschein	+
Saponinas	Espuma	+
Cumarinas	Hidroxilamina	-
Naftoquinonas	Bornträger	-
Antraquinonas	Bornträger	-
Glucósidos cardiotónicos	Kedde	-
Sesquiterpenlactonas	Bajlet	-
Leucoantocianinas	Rosenheim	-

- : ausencia del metabolito, +: presencia del metabolito

Las cumarinas, naftoquinonas, antraquinonas, glucósidos cardiotónicos, sesquiterpenlactonas y leucoantocianinas no se encontraron en el extracto etanólico.

### Actividad antifúngica in vitro

Se evaluó la actividad antifúngica *in vitro* mediante el método de difusión en cultivo de agar frente a microorganismos *Candida albicans* ATCC 10231, *Aspergillus brasiliensis* ATCC 16404 y *Trichophyton mentagrophytes* ATCC 9533 y se demostró la actividad a través de los halos de inhibición. (Figura 2).

**Figura 2:** Experimento representativo que muestra la comparación de los halos de inhibición de cada microorganismo: **A)** *Candida albicans *ATCC 10231, **B)** *Aspergillus brasiliensis *ATCC 16404 y **C)** *Trichophyton mentagrophytes *ATCC 9533 frente al extracto etanólico de las hojas de *Solanum hispidum *Pers y los antifúngicos estándar (controles positivos)

La Tabla 3, presenta los resultados de la actividad antifúngica de las hojas* Solanum hispidum* Pers. El ensayo demostró que para *C. albicans* el halo de inhibición fue de 26 mm (± 0,38), para *A. brasiliensis *se obtuvo un halo de 23 mm (± 0,53) y *T. mentagrophytes* reveló un halo de inhibición de 25 mm (± 1,31). De todos ellos, el extracto etanólico de las hojas de *Solanum hispidum* Pers presentó mayor actividad para *C. albicans*, sin embargo, su control positivo (nistatina) presentó un halo mayor (30 mm) en comparación al extracto etanólico.

**Tabla 3:** Actividad antifúngica del extracto etanólico de las hojas de *Solanum hispidum* Pers frente a los microorganismos.

Microorganismo	Extracto etanólico (mg/mL)	Diámetro del halo de inhibición mm ±DS
*Candida albicans* ATCC 10231	25 mg/mL Nistatina (2mg/mL)	26 ± 0,38 30 ± 0,38
*Aspergillus brasiliensis* ATCC 16404	25 mg/mL Fluconazol (2mg/mL)	23 ± 0,53 15 ± 0,53
*Trichophyton mentagrophytes* ATCC 9533	25 mg/mL Ketonazol (2mg/mL)	25 ± 1,31 24 ± 0,76

DS: desviación estándar

### Determinación de la concentración mínima inhibitoria (CMI)

Posterior a la demostración de actividad antifúngica, se realizó la CMI. Los resultados muestran que la concentración más baja del extracto etanólico de las hojas de *Solanum hispidum* Pers que inhibe completamente el crecimiento para *C. albicans* fue de 125 µg/mL; para *A. brasiliensis* fue de 250 µg/mL y para *T. mentagrophytes* fue de 125 µg/mL; en base al criterio de actividad antifúngica, todas ellas demostraron actividad moderada (Tabla 4).

**Tabla 4:** Valores de la concentración mínima inhibitoria (CMI) (µg/mL) del extracto etanólico de las hojas de *Solanum hispidum *Pers frente a los microorganismos.

Microorganismo	Concentración mínima inhibitoria (µg/mL)	Criterio de actividad antifúngica
*Candida albicans* ATCC 10231	125	Moderada
*Aspergillus brasiliensis* ATCC 16404	250	Moderada
*Trichophyton mentagrophytes* ATCC 9533	125	Moderada

## DISCUSIÓN

El presente estudio determinó que el extracto etanólico estabilizado de las hojas de *Solanum hispidum* Pers presenta una mayor solubilidad frente a etanol y metanol, es decir, con tendencia a solventes polares, ambos son usados ampliamente; sin embargo, en este estudio se continuó con etanol debido a la disponibilidad, considerando, además, que la mayoría de metabolitos con actividad antifúngica poseen polaridad intermedia y pueden concentrarse fácilmente en este tipo de solventes ^26^. Nuestros resultados son concordantes con otros estudios realizados en extractos crudos ^24^.

Los análisis cualitativos fueron desarrollados para detectar los metabolitos presentes en el extracto etanólico de las hojas de *Solanum hispidum* Pers, los resultaron evidenciaron la presencia de múltiples metabolitos como compuestos fenólicos, taninos, flavonoides, esteroides, alcaloides y saponinas; además, para asegurar su presencia inequívoca se usaron cuatro pruebas diferentes para alcaloides y cuatro pruebas diferenciales para esteroides con respecto a los triterpenoides ^18^, demostrando así la confiabilidad de los resultados.

En este sentido, se ha reportado que el género *Solanum* presenta abundancia de alcaloides y esteroides tal es el caso se *Solanum chrysotrichum* ^6^ ^,^ ^7^ *, Solanum xanthocarpum* ^27^ *, Solanum nigrum* ^11^ *, Solanum surattense* ^28^ y* Solanum quitoense* ^29^. Retamozo ^14^ logró identificar glicoalcaloides esteroidales a través de pruebas cualicuantitativas en la misma especie *Solanum hispidum* Pers; además, analizó el contenido en hojas y frutos, demostrando un mayor contenido en el fruto a comparación de las hojas; también se explica que la variabilidad del contenido está influenciada por diferentes factores como estado vegetativo, época de colecta, procedencia, etc. Por lo tanto, se confirma la presencia de alcaloides y esteroides como componentes del extracto de las hojas de* Solanum hispidum* Pers.

Se empleó la concentración del extracto a 25 mg/mL en DMSO, en base a estudios previos de tamizaje de extractos etanólicos de plantas peruanas con actividad antifúngica; Rojas *et al*. ^19^ y Quiroz ^21^obtuvieron resultados favorables utilizando esa concentración en 24 y 8 plantas medicinales, respectivamente.

Respecto a la actividad antifúngica, el extracto etanolico de las hojas* Solanum hispidum* Pers mostró un efecto inhibitorio sobre el crecimiento de *C. albicans*, *A. brasiliensis* y *T. mentagrophytes* con zonas de inhibición entre 23 a 26 mm. En este sentido, Rojas *et al.* ^19^ mencionaron que la actividad antifúngica con halos de inhibición superiores a 18 mm, utilizando el método de difusión en pozo de agar, es un indicador del buen desempeño como potencial agente terapéutico.

La investigación realizada por Das *et al.* ^8^ demostró halos de 18 mm contra el patógeno* C. albicans* empleando *Solanum melongena*; por su parte Shubha *et al*. ^27^ , reportaron halos de 12 mm empleando extracto de *Solanum xantocarpum*; ambas especies pertenecen de la familia *Solanaceae. E*n estudios previos, las especies *Solanum nigrum* y *Solanum Xanthocarpum* también demostraron actividad frente a *C*.* albicans* mediante el método de difusión en disco ^26^. Además, un estudio de tamizaje de plantas peruanas reveló que una especie de la familia Solanaceae demostró mayor actividad, evidenciando zonas de inhibición de 19 mm con el método de difusión en pozo de agar ^19^. Los resultados de esta investigación evidencian halos superiores de inhibición (≥ 23 mm) en comparación a los estudios frente a otras especies del género *Solanum* ^(8, 19, 26, 27)^.

Así también, el presente estudio demostró una actividad antifúngica moderada para *C. albicans *con valores de 125 µg/mL, estos valores son menores a lo reportado en la especie de *Solanum mammosum *con valores de 256 µg/mL ^30^. También se ha reportado actividad antifúngica frente a otras especies fúngicas como *Aspergillus *sp; como *Solanum xantocarpum* contra *A. niger* ^10^, mostrando actividad antifúngica con CMI de 250 µg/mL, estos valores también han sido hallados en nuestro estudio (250 µg/mL) frente a *A. brasiliensis*. Para el caso de *Trichophyton mentagrophytes *se evidenció la CMI de 125 µg/mL, resultados similares se reportaron con *Solanum mammosum* con valores de 256 µg/mL ^30^ para *T. mentagrofphytes*.

Muchas especies de la familia Solanaceae como *Solanum chrysotrichum* ^6^^,^^7^*, Solanum melogena* ^8^, *Solanum nigrum* ^11^, *Solanum xanthocarpum* ^23^ y *Solanum mammosum* ^30^ también demostraron actividad antifúngica, es posible que esta se deba a la presencia de saponinas, alcaloides, esteroides y/o flavonoides, los cuales pueden actuar de manera individual o sinérgica por un mecanismo de acción aún desconocido.

Tanto los esteroides como los alcaloides presentan alta actividad biológica y son un grupo de compuestos cíclicos que han sido estudiados debido a sus efectos antimicrobianos, esto ha sido reafirmado a través del aislamiento de compuestos bioactivos con potente actividad antifúngica *in vitro* ^30^.

Una de las limitaciones de nuestro estudio es que se utilizó exclusivamente las hojas y no los frutos, que también son usados en las zonas aledañas donde se recolectaron los especímenes. Esto se debe a que no son renovables y se prefirió no afectar su crecimiento natural y habitual; no obstante, se plantea en una siguiente etapa complementar con el fraccionamiento y la caracterización de los compuestos bioactivos de estos frutos, complementando así el estudio.

El presente estudio *in vitro* presenta un análisis fitoquímico preliminar del extracto de *Solanum hispidum* Pers., durante el cual se logró identificar los metabolitos secundarios principales, información no conocida previamente, además se demostró la actividad moderada antifúngica *in vitro* del extracto etanólico de las hojas de *Solanum hispidum* Pers.
